# Treating impulsivity with probiotics in adults (PROBIA): study protocol of a multicenter, double-blind, randomized, placebo-controlled trial

**DOI:** 10.1186/s13063-019-4040-x

**Published:** 2020-02-11

**Authors:** Gara Arteaga-Henríquez, Silvia Karina Rosales-Ortiz, Alejandro Arias-Vásquez, Istvan Bitter, Ylva Ginsberg, Pol Ibañez-Jimenez, Tünde Kilencz, Catharina Lavebratt, Silke Matura, Andreas Reif, Janos Rethelyi, Vanesa Richarte, Nanda Rommelse, Anne Siegl, J. Antoni Ramos-Quiroga

**Affiliations:** 1 Department of Psychiatry, Hospital Universitari Vall d’Hebron, Barcelona, Catalonia Spain; 2 Department of Human Genetics, Donders Institute for Brain, Cognition and Behavior, Radboud University Medical Centre, Nijmegen, The Netherlands; 3 Department of Psychiatry, Donders Institute for Brain, Cognition and Behavior, Radboud University Medical Centre, Nijmegen, The Netherlands; 4 Department of Psychiatry and Psychotherapy, Semmelweis University, Budapest, Hungary; 5 Department of Clinical Neuroscience, Centre for Psychiatry Research, Karolinska Institute, Stockholm, Sweden; 6 Department of Molecular Medicine and Surgery, Karolinska Institute, Stockholm, Sweden; 7 Center for Molecular Medicine (CMM), Karolinska University Hospital, Stockholm, Sweden; 8 Department of Psychiatry, Psychosomatic Medicine and Psychotherapy, University Hospital, Goethe University, Frankfurt, Germany; 9 Group of Psychiatry, Mental Health and Addictions, Vall d’Hebron Research Institute (VHIR), Barcelona, Catalonia Spain; 10 Biomedical Network Research Centre on Mental Health (CIBERSAM), Barcelona, Catalonia Spain; 11 Department of Psychiatry and Legal Medicine, Universitat Autònoma de Barcelona, Barcelona, Catalonia Spain; 12 Karakter Child and Adolescent Psychiatry University Centre, Nijmegen, The Netherlands

**Keywords:** Impulsivity, Compulsivity, Aggression, Microbiome, Probiotics, Synbiotics, ADHD, Borderline personality disorder, Nutrition

## Abstract

**Background:**

Impulsivity and compulsivity are related to emotional and social maladjustment and often underlie psychiatric disorders. Recently, alterations in microbiota composition have been shown to have implications for brain development and social behavior via the microbiota–gut–brain axis. However, the exact mechanisms are not fully identified. Recent evidence suggests the modulatory effect of synbiotics on gut microbiota and the contribution of these agents in ameliorating symptoms of many psychiatric diseases. To date, no randomized controlled trial has been performed to establish the feasibility and efficacy of this intervention targeting the reduction of impulsivity and compulsivity. We hypothesize that supplementation with synbiotics may be an effective treatment in adults with high levels of impulsivity and/or compulsivity.

**Methods/design:**

This is a prospective, multicenter, double-blind, randomized controlled trial with two arms: treatment with a synbiotic formula versus placebo treatment. The primary outcome is the response rate at the end of the placebo-controlled phase (response defined as a Clinical Global Impression–Improvement Scale score of 1 or 2 = very much improved or much improved, plus a reduction in the Affective Reactivity Index total score of at least 30% compared with baseline). A total of 180 participants with highly impulsive behavior and a diagnosis of attention deficit/hyperactivity disorder (ADHD) and/or borderline personality disorder, aged 18–65 years old, will be screened at three study centers. Secondary outcome measures, including changes in general psychopathology, ADHD symptoms, neurocognitive function, somatic parameters, physical activity, nutritional intake, and health-related quality of life, will be explored at assessments before, during, and at the end of the intervention. The effect of the intervention on genetics, microbiota, and several blood biomarkers will also be assessed. Gastrointestinal symptoms and somatic complaints will additionally be explored at 1-week follow-up.

**Discussion:**

This is the first randomized controlled trial to determine the effects of supplementation with synbiotics on reducing impulsive and compulsive behavior. This clinical trial can contribute to explaining the mechanisms involved in the crosstalk between the intestinal microbiome and the brain. If effects can be established by reducing impulsive and compulsive behavior, new cost-effective treatments might become available to these patients.

**Trial registration:**

ClinicalTrials.gov, NCT03495375. Registered on 26 February 2018.

## Background

Impulsivity may be defined as “a predisposition towards rapid, unplanned reactions to internal or external stimuli, with diminished regard to the negative consequences that such reactions may have for the impulsive individual or others” [1–4]. In contrast, compulsivity represents “the performance of repetitive and functionally impairing overt or covert behavior without an adaptive function, performed in a habitual or stereotyped fashion, either according to rigid rules or as a means of avoiding perceived negative consequences” [2, 5]. Both traits share neuronal mechanisms involving a dysfunctional inhibition of thoughts and behavior [1, 6], and, rather than unitary phenomena, they are considered as multidimensional constructs that involve disruption within a range of neural processes, including attention, perception, and coordination of motor and/or cognitive processes. Impulsive and compulsive symptoms are overrepresented in individuals with several psychiatric disorders, such as attention-deficit/hyperactivity disorder (ADHD), borderline personality disorder (BPD), and/or obsessive-compulsive disorder [7]. Importantly, maladaptive impulsivity and compulsivity can lead to serious consequences not only for affected individuals and their families but also for society. They predispose individuals to aggressive or addictive behaviors, increasing the risk for mortality [8]. Despite this, data about modifiable risk and protective factors are largely lacking.

### Nutrition, brain, and behavior

Not only heritability, sex, and socioeconomic status (SES) but also diet may play a pivotal role in impulsive and compulsive symptomatology [9, 10]. In line with this, a number of studies have shown that different nutrient combinations may have interactive effects on cognitive function and behavior, including antioxidants; omega-3 polyunsaturated fatty acids (PUFAs); monounsaturated fatty acids; polyphenols; potassium; calcium; zinc; fiber; folate; and/or vitamins A, B_12_, C, D, or E [11–13]. More specifically, recent reports have linked aggressive behavior with low blood omega-3 PUFA levels and/or with low seafood consumption [14], and several animal studies have found that the long-term consumption of a low-calorie diet enhances autophagy and protects neurons effectively against aging, maintaining learning and memory capacity, whereas long-term consumption of a high-calorie diet facilitates neuronal loss in the hippocampus, aggravating age-related cognitive impairments [15, 16].

Importantly, the type of food intake can influence brain development and function in all age groups, with a recent study showing that prenatal exposure to an unhealthy diet was associated with ADHD symptoms, further linked to altered epigenetic modification of blood-derived DNA [17, 18]. However, these findings have not been consistently replicated in large-sample randomized controlled trials (RCTs), and the underlying mechanisms remain unknown [19–23].

### Gut microbiome, microbiota, and behavior

Although *microbiota* refers to the specific microorganisms that are found within a specific environment, *microbiome* refers to the collection of genomes from all the microorganisms found in this particular environment [24]. It is hypothesized that an imbalance in the gut microbiota (dysbiosis) may have a negative effect on neurodevelopment, behavior, and cognition [25–31]. Related to this, changes in human microbiome and/or microbiota composition have been consistently found in individuals with autism spectrum disorder (ASD) [32–36], and a pilot study on the microbiome has demonstrated, for the first time, a difference in several bacterial taxa between subjects with ADHD and healthy control subjects [37]. Specifically, lower *Firmicutes* genus and higher *Bifidobacterium* genus were found in subjects with ADHD than in healthy individuals, with the increase in the *Bifidobacterium* genus relating to decreased ventral striatal functional magnetic resonance imagining responses during reward anticipation [37].

The relationship between gut microbiota and the brain seems to be bidirectional. The gut microbiota modulates brain function and development, and the brain can alter the gut microbiota, allowing colonization by pathogenic bacteria [38–40]. Illustrating this idea, some studies have shown an overrepresentation of gastrointestinal symptoms in patients with both neurodevelopmental and neuropsychiatric disorders [41–48], and a study in healthy students found that during exams, psychological stress increased at the same time that numbers of lactobacilli in stool samples decreased [49].

But how does this bidirectional communication—the gut–brain axis—work? The “enteric nervous system” is complex and, regarding neurotransmitters and signaling molecules, similar to the brain [50]. As an example, 95% of all circulating serotonin, dopamine, or noradrenaline precursors are produced by our gut microbiota [50]. This system is connected to the central nervous system through hormonal or innate neuronal pathways [51] that are critical for its development and vice versa [52]. Furthermore, studies have shown that the gut flora is critically involved in immunoregulation [53–55], whereas some reports have shown the immune system as an important regulator of neurodevelopment and synaptic function in the brain [56]. In line with this, immune dysfunction and/or autoimmunity have been speculated to be associated with many neuropsychiatric and neurodevelopmental disorders, such as ADHD [57, 58]. Supporting this idea, an increased incidence of immune-mediated disorders (e.g., asthma, allergic rhinitis, atopic dermatitis, allergic conjunctivitis, psoriasis, thyrotoxicosis, or type 1 diabetes) has been found among patients with ADHD [59–62]. Moreover, elevated inflammatory markers (especially interleukin [IL]-6) [63–65] or autoantibody levels (e.g., anti–basal ganglia antibodies, antibodies against the dopamine transporter) [66, 67] have been found both in serum/plasma and in cerebrospinal fluid of these patients [63, 68, 69].

### Probiotic/synbiotic interventions

Probiotic bacteria, or “probiotics,” are live, nonpathogenic microorganisms that normally inhabit the intestines and contribute to the health of the host by improving microbial balance [70]. On the other hand, prebiotics are nondigestible ingredients that selectively stimulate the growth and activity of these probiotic microorganisms [71]. The synergic combination of probiotics and prebiotics is referred to as *synbiotics*. Recent findings suggest that probiotics and/or synbiotics can not only revert dysbiosis but also modulate brain activity and improve cognition, mood, and behavior [72–78]. Importantly, a recent study has shown that oral administration with *Lactobacillus* during the first 6 months of life reduced the prevalence of ADHD or ASD at the age of 13 [79]. However, the exact mechanisms by which probiotics and/or synbiotics exert their action remain unknown. Recent findings suggest immunomodulatory and anti-inflammatory properties of these agents [80], possibly by selectively targeting T-helper (Th) type 1 [81] and Th17 cell lineages [82].

The aim of the present multicenter, prospective, double-blind, placebo-controlled, parallel-group study is to investigate the effect of a synbiotic formula (Synbiotic 2000 Forte 400; Synbiotic AB, Höganäs, Sweden) on reducing impulsive, compulsive, and aggressive behaviors in a sample of highly impulsive adults with a diagnosis of attention-deficit/hyperactivity disorder (ADHD) and/or borderline personality disorder (BPD). In this study, we will test the idea that supplementation with probiotics will, by modifying the gut microbiota structure and metabolism, reduce impulsive, compulsive, and aggressive behaviors in this specific population and thereby improve their daily life function. Moreover, we will evaluate the composition of gut microbiota in this population and link it to inflammatory/immunological abnormalities that can underlie core symptoms of these disorders.

## Methods/design

This protocol is presented in accordance with the 2013 SPIRIT (Standard Protocol Items: Recommendations for Interventional Trials) Statement (See Additional file 1 for the populated SPIRIT Checklist (83).

### Trial design and study setting

The Treatment of impulsivity in adults with probiotics (PROBIA) trial is designed as a 10-week multicenter, prospective, randomized, double-blind, placebo-controlled, parallel design study. It will be performed in close cooperation between three European clinical centers: Goethe University Hospital Frankfurt (Department of Psychiatry, Psychosomatic Medicine and Psychotherapy), Frankfurt, Germany; Vall d’Hebron Research Institute (Psychiatry, Mental Health and Addictions Group), Barcelona, Spain; and Semmelweis University (Department of Psychiatry and Psychotherapy), Budapest, Hungary. These centers are all affiliated with or part of university hospitals and, with around 300 new inpatients and outpatients visiting each center every year, are considered as reference centers for the treatment of ADHD, BPD, and other disorders characterized by high impulsivity levels.

### Study population and recruitment

Participants will be eligible for participation in this study if they meet all the inclusion criteria and none of the exclusion criteria listed in Table 1. With the aim of gender balancing the study, at least 30% female participants will be included.

**Table 1:** Inclusion and exclusion criteria of the PROBIA trial

Inclusion criteria	Both males and females aged 18–65 years
	A high level of multidimensional impulsivity based on both a Clinical Global Impression–Severity Scale (CGI-S) score ≥ 4 and an Affective Reactivity Index (ARI) ≥ 5
	DSM-5 criteria for attention deficit/hyperactivity disorder (ADHD) and/or borderline personality disorder (BPD) confirmed by a structured diagnostic interview (ADHD: Diagnostic Interview for Adult ADHD [DIVA 2.0]; BPD: Structured Clinical Interview for DSM-IV [SCID-II])
	Deemed reliable and compliant with the protocol by the investigator
	Ability to speak and comprehend the native language of the country in which the assessments take place
	Informed consent signed
Exclusion criteria	Antibiotherapy within the last 6 weeks prior to study
	Currently taking probiotics
	Presence of a major psychiatric disorder with psychotic symptoms or other major psychiatric conditions requiring hospitalization (e.g., significant mood disorders)
	Neurological disorders involving central functions (e.g., epilepsy, multiple sclerosis, narcolepsy)
	Intelligence quotient (IQ) < 70 (measured by WAIS, if available)
	Major physical illnesses of the cardiovascular, endocrine, pulmonal, immune, or gastrointestinal system or undergoing immunosuppression
	History of/present clinically relevant somatic acute or chronic disorders that, in the opinion of the investigator, might confound the results of tolerability/safety assessments or prohibit the patient from completing the study or would not be in the best interest of the patient
	Documented allergy, hypersensitivity, or intolerance to any of the ingredients of the intervention
	Subject has taken another investigational product or taken part in a clinical study within 30 days prior to entering the study.

*Abbreviations: DSM Diagnostic and Statistical Manual of Mental Disorders*, *PROBIA* Treatment of impulsivity in adults with probiotics, *WAIS* Wechsler Adult Intelligence Scale

**Fig. 1:**
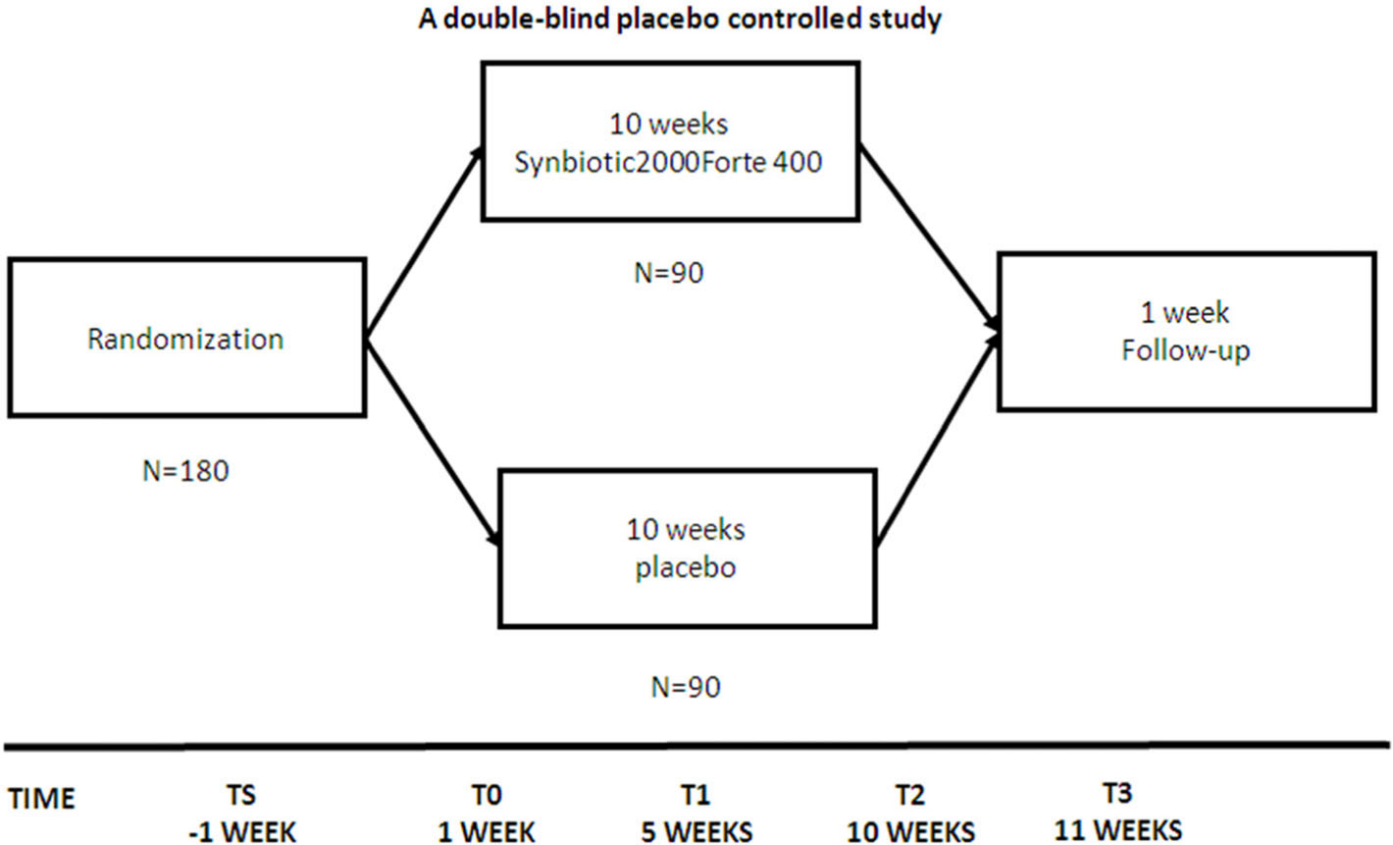
Timeline of the PROBIA study

Recruitment began on 22 February 2019 and will have a duration of 2 years. A total of 180 participants (60 subjects per site) will be recruited by trained psychiatrists and/or psychologists at the different sites (see Fig. 1). Strategies aimed at optimizing the recruitment process will include the distribution of handouts or flyers to colleagues, physicians, and families, as well as an offer of reimbursement of study-related travel costs to participants. Furthermore, and depending on local regulations, information via presentations, websites, and social media campaigns will be provided. Regular calls will be made to patients to avoid participant withdrawal from the study.

### Randomization and study intervention

After eligibility checks have been conducted and written consent has been obtained by the different psychiatrists and/or psychologists of the trial team, eligible participants will be randomized to the experimental (EG) or the control (CG) group in a 1:1 allocation ratio using an independent web-based computerized service (www.randomization.com). Randomization will be center-stratified and have a fixed length per center, being independent of participant characteristics. However, due to the large sample size, a balanced ratio of baseline characteristics between the EG and CG is expected.

Individual participant treatment kits will contain all required daily synbiotic or placebo envelopes for the 10-week RCT and will be prepared in advance by sequentially numbering and labeling them as “A” or “B.” Because this is a double-blind study, neither the participants nor the clinicians involved in the trial will have access to the randomization list.

Synbiotic 2000 Forte 400 (SF) is a synbiotic formula currently produced by Synbiotic AB in Höganäs, Sweden. Each dose (powder-containing sachet) of SF contains 100 billion of each of *Pediococcus pentosaceus* 5-33:3, *Lactobacillus paracasei subsp. paracasei* 19, *Lactobacillus plantarum* 2362, and *Leuconostoc mesenteroides* 77:1, in combination with four bioactive fermentable fibers (2.5 g each of β-glucan, inulin, pectin, and resistant starch). SF is categorized as a generally regarded as safe agent, being tested on several hundreds of critically ill patients (e.g., pancreatitis, liver transplant) with no side effects or specific and/or relevant risks conferred to the trial participants [83]. The placebo (also provided by Synbiotic AB) is composed of the polysaccharide maltodextrin and has a texture and flavor similar to SF. Participants will be asked to continue with their previous medications and/or usual treatments and add the synbiotic or placebo once daily on top of cold foods such as yogurt, muesli, or salad. To secure bacterial viability, participants will be asked to store both SF and placebo at + 4–6 °C.

### Outcome measures

#### Primary outcomes

The primary outcome measure will be response to treatment, defined by a reduction in the Affective Reactivity Index–Self-Report (ARI-S) scale total score of at least 30% compared with baseline plus a Clinical Global Impression–Improvement Scale total score of 1 or 2 (very much improved or much improved) at the end of the placebo-controlled phase.

#### Secondary outcomes

The secondary outcome measures are selected to assess intervention effects on symptoms of impulsivity, compulsivity, and aggression via a series of selected scales and questionnaires (e.g., the Strengths and Difficulties Questionnaire, Yale-Brown Obsessive Compulsive Scale, Difficulties in Emotion Regulation Scale, and the Urgency, Premeditation [lack of], Perseverance [lack of], Sensation Seeking, Positive Urgency, Impulsive Behavior Scale [UPPS-P]). Interestingly, the UPPS-P consists of a 59-item self-report assessing five subscales (negative and positive urgency, premeditation, perseverance, and sensation seeking), with a mean value being calculated separately for each scale, allowing the estimation of distinct dimensions of impulsive behavior [84]. Another secondary outcome measure will be the change in ADHD symptoms (via the ADHD Rating Scale [ADHD-RS]). Like the UPPS-P, the ADHD-RS provides not only a total ADHD score but also separate scores for inattentive, hyperactive, and impulsive subscales, providing a better characterization of patients.

Changes in general psychopathology, major stressors, neurocognitive function, sleep disturbances, body-related measures (e.g., pulse rate, blood pressure, body mass index, waist–hip circumference ratio), medications taken, physical activity (via mobile health application and movement sensors), health-related quality of life, microbiome composition, and blood markers will also be evaluated. Blood markers will include genetic parameters, hormones, neurotransmitters, nutrients, and inflammatory/anti-inflammatory molecules (see Table 2).

**Table 2:** Measurement of blood markers

Type of marker	Marker name	Link with:	Tissue
Inflammation	TNF-α	Inflammation/exercise*/diet	Serum/plasma
	IL-1β	Inflammation/exercise*/diet	Serum/plasma
	IL-6	Inflammation/exercise*/diet	Serum/plasma
	CRP	Inflammation/diet	Serum/plasma
	Bacterial lipoprotein	Inflammation/diet	Serum/plasma
	Vitamin B_12_	Diet	Serum/plasma
	Folic acid	Diet	Serum/plasma
Vitamins and minerals	Vitamin D	Diet	Serum/plasma
	Iron	Diet	Serum/plasma
	Cu	Diet	Serum/plasma
Nutrients	Cholesterol	Diet	Serum/plasma
	Glucose	Diet	Serum/plasma
	Homocysteine	Diet	Serum/plasma
	BDNF	Diet	Serum/plasma
	5-HT	Diet	Serum/plasma
Other markers	SCFA	Diet	Feces
	GLP-1	Hormone-diet	Serum/plasma
	Insulin	Hormone-diet	Serum/plasma
	Paraoxonase	Diet	Serum/plasma

*Abbreviations: 5-HT* serotonin, *BDNF* brain-derived neurotrophic factor, *CRP* C-reactive protein, *GLP-1* glucagon-like peptide 1, *IL* interleukin, *SCFA* short-chain fatty acid, *TNF-α* tumor necrosis factor-α

Blood samples (34.5 ml) will be fractioned into serum and plasma and stored at − 80 °C. Then, they will be shipped to the Department of Human Genetics of Radboudumc, Nijmegen, The Netherlands, for storage. Thereafter, blood markers will be measured at the Department of Clinical Biochemistry at Radboudumc in Nijmegen according to validated protocols. Participants willing to take part in the assessment of their microbiome will receive a specially designed container for feces collection, allowing participants to send the samples by ordinary post to the individual centers, where they will be processed and stored at − 80 °C for later shipping to the Department of Human Genetics of Radboudumc for sequencing and analysis of bacterial DNA. Crucially, before and during the intervention, the participants will be asked to send back their fecal samples within 24 h after the test sessions.

Another secondary outcome measure will be the change in nutritional intake. Participants will be asked to complete at least three 24-h dietary recalls (24HDRs): at baseline, during the intervention, and directly afterward, including two weekdays and one weekend day on nonconsecutive days. They will need to describe the type and amount (in grams) of all drinks and foods consumed during the previous days, starting with the first intake after waking up in the morning. Moreover, they will have to specify the time for every meal occasion during the day (breakfast, lunch, dinner, or snack).

Participant compliance with the intervention (SF or placebo) will be monitored with the Probabilistic Medication Adherence Scale throughout the 10 weeks of the intervention. Moreover, participants will be asked to return unused envelopes from the previous visit. This will enable us to secure bacterial viability and at the same time estimate adherence to treatment. Finally, the presence of somatic complaints/side effects or gastrointestinal symptoms (via the Bristol Stool Scale) will also be evaluated.

### Participant timeline

The trial timeline is shown in Table 3. Time points per intervention will be selected on the basis of known duration to exert effect.

**Table 3:** Standard Protocol Items: Recommendations for Interventional Trials (SPIRIT) schematic schedule of enrollment, interventions, and assessments

Study procedures		Screening	Open label			Follow-up
	Visit	TS	T0	T1	T2	T3
	Week	−1	1	5	10	11
	Day	−7 to −1	1	35 ± 2	70 ± 2	77 ± 2
Informed consent form		X				
Inclusion/exclusion criteria		X				
Demographics (including SES)		X				
IQ (IQ score based on WAIS, if available)		X				
M.I.N.I.-Plus assessment		X				
Confirmation of diagnosis/-es		X				
Confirmation of ADHD	DIVA 2.0	X				
Confirmation of BPD	SCID-II	X				
Primary outcome						
Clinical Global Impression–Improvement Scale	CGI-I			X	X	X
Affective Reactivity Index	ARI-S	X	X	X	X	X
Secondary outcomes						
Clinical Global Impression–Severity Scale	CGI-S	X	X	X	X	
Clinician rating of ADHD symptoms	ADHD-RS		X	X	X	
Self-rating of multidimensional impulsivity	UPPS-P		X	X	X	
Questionnaire on well-being			X		X	
Functioning Assessment Short Test	FAST		X	X	X	
Self/other rating of strength and difficulties	SDQ		X	X	X	
Clinician rating of compulsivity	Y-BOCS		X	X	X	
Sleep problems	5-item questionnaire		X	X	X	
Self-rating of emotion regulation difficulties	DERS-16		X	X	X	
Somatic complaints/side effects	Body system questions			X	X	X
Gastrointestinal symptoms	Bristol Stool Scale		X	X	X	X
Self-rating of perceived stress	PSS		X	X	X	
Major stressors			X	X	X	
Blood pressure, pulse rate, height, weight, waist, hip, medical history, medication evaluation	Assessed by clinician		X	X	X	
Physical activity	Actigraphy; mobile health application		X		X	
Blood biomarkers	Blood sample		X	X	X	
Microbiome	Stool sample		X	X	X	
DNA and RNA	Blood sample		X	X	X	
Neurocognitive measures	CPT-II		X	X	X	
Nutritional intake	Food diary		X	X	X	
Treatment adherence/satisfaction/beliefs						
Probabilistic Medication Adherence Scale	ProMAS		X	X	X	

*Abbreviations: ADHD* attention-deficit/hyperactivity disorder, *ADHD-RS* Attention-Deficit/Hyperactivity Disorder Rating Scale, *ARI-S* Affective Reactivity Index–Self-Report, *BPD* borderline personality disorder, *CGI-I* Clinical Global Impression–Improvement Scale, *CGI-S* Clinical Global Impression–Severity Scale, *CPT-II* Continuous Performance Test, *DERS-16* Difficulties in Emotion Regulation Scale, *DIVA 2.0* Diagnostic Interview for Adult ADHD, Second Edition, *FAST* Functioning Assessment Short Test, *IQ* intelligence quotient, *M.I.N.I.-Plus* Mini-International Neuropsychiatric Interview, *PROBIA* Treatment of impulsivity in adults with probiotics, *ProMAS* Probabilistic Medication Adherence Scale, *PSS* Perceived Stress Scale, *SCID-II* Structured Clinical Interview for DSM-IV Axis II Disorders, *SDQ* Strengths and Difficulties Questionnaire, *SES* socioeconomic status, *UPPS-P* Urgency, Premeditation (lack of), Perseverance (lack of), Sensation Seeking, Positive Urgency, Impulsive Behavior Scale, *WAIS* Wechsler Adult Intelligence Scale, *Y-BOCS* Yale-Brown Obsessive Compulsive Scale

At the screening visit (TS), the rate of impulsivity will be established by performing structured interviews, the ARI-S, and the Clinical Global Impression–Severity Scale. After having established the presence of high impulsivity, the diagnosis of ADHD and/or BPD will be assessed via the Diagnostic Interview for Adult ADHD, Second Edition, and the Structured Clinical Interview for DSM-IV Axis II Disorders section for BPD, respectively. Thereafter, screening for eligibility based on clinical history, demographics (including SES), tests, and questionnaires (e.g., Mini-International Neuropsychiatric Interview [M.I.N.I.-Plus], IQ tests [Wechsler Adult Intelligence Scale, Fourth Edition]) will be performed, and informed consent will be obtained. Once this process is finished, a mobile health system as well as a food record will be introduced to participants (duration, approximately 1 h). The mobile health system will enable patients to share (via a mobile application) data of interest regarding their physical activity (acceleration, number of steps).

Trial-specific assessments will be done at T0 (duration, approximately 3 h), scheduled within 1 week after TS. During the week between TS and the baseline visit (T0), the mobile health system will be used at home. Here and during 24 h, participants will wear movement sensors on two working days and on Saturday and Sunday, and they will be asked to complete three 24HDRs on a web-based tool or paper on two nonconsecutive weekdays and one weekend day (duration, 15 min each). Individuals will also be asked to collect feces on one day during the 1-week period at home and to bring it along at T0.

In addition to responding to the questionnaires and scales listed in Table 1, participants will be instructed on how to store and eat the study product (SF or placebo). The intervention will begin on the day after T0, and participants will be asked to continue with their usual medications and/or treatments. Participants will have to complete another three 24HDRs, collect their feces before the next visit, and bring it along at T1, following the same guidelines as during the screening assessments.

T1 (midintervention assessment; duration, approximately 3 h) will take place 5 weeks after T0. This midintervention assessment aims at obtaining the primary and secondary outcome measures during the ongoing trial. Participants will be asked once again to wear movement sensors, complete another three 24 HDRs, and collect their feces 1 week before T2 and bring it along at T2. At 10 weeks after initiating the probiotic or placebo intervention (T2), primary and some secondary outcome measures will be assessed again (duration, approximately 3 h).

Within 1 week after T2 and with the aim of measuring safety aspects of the intervention (i.e., somatic complaints/side effects as well as gastrointestinal symptoms), the follow-up (T3) assessment will take place (duration, approximately 20 min).

### Criteria for discontinuing allocated interventions

Any undesirable experience (of either a physical or psychological nature) occurring to a participant during the study, whether or not considered related to undergoing treatment with SF or placebo, will be considered an adverse event (AE). Participants will be asked for AEs at each visit and then will be discussed with the study physician at the site. Thus, if an AE occurs in response to SF or placebo, this will immediately be recorded by the principal investigator (PI) or the PI’s staff, and the participant may be discontinued from the study.

Serious adverse events (SAEs), serious adverse reactions (SARs) or unexpected serious adverse reactions are defined, respectively, as any adverse event, reaction, or unexpected adverse reaction that results in death; is life-threatening and/or requires hospitalization or prolongation of existing hospitalization; results in persistent or significant disability or incapacity; or consists of a congenital anomaly or birth defect. Any of them will be communicated immediately to the lead coordinator of the trial at Vall d’Hebron Research Institute (VHIR) and to the coordinating PI at the clinical site, who will be responsible for reporting the event to the ethics committee that approved the protocol. Suspected unexpected serious adverse reactions (SUSARs) that are fatal or life-threatening must also be reported not later than 7 days after the sponsor is first made aware of the reaction. Any additional relevant information must be reported within 8 days. SUSARs that are not fatal or life-threatening must be reported within 15 days of the sponsor first becoming aware of the reaction. All ongoing AEs will be followed up until no more signs and symptoms are verifiable or until stability has been reached. Depending on the event, additional tests or medical procedures, as well as referrals to a general physician or a medical specialist, will be indicated during this follow-up phase.

In the case that a participant’s psychological state deteriorates to a clinically significant degree during the trial, the investigators will either discuss with the participant the possibility of withdrawing from the study or decide that the participant should withdraw. If a participant, for any reason, requires treatment with certain therapeutic agents (i.e., antibiotics), the agent taken and time of administration will be noted. If any other protocol exclusion violation has occurred, the participant’s involvement will be discontinued. Participants may at any time request to be withdrawn from the study or revoke their consent to participate. If a patient is discontinued from the trial, a follow-up visit will be carried out to ensure the well-being of the participant.

### Data management

Participants and clinical staff will be able to fill out all requested questionnaires online via the Castor database system (http://castoredc.com/). Then, data will be directly uploaded to a central database using a macro software. Both the Castor database system and macro software are fully secure and in accordance with current standards (i.e., Good Clinical Practice [GCP], 21 CFR Part 11, EU Data Protection Directive, ISO27001 and ISO9001 hosting). Both systems are also approved by the Clinical Research Centre Nijmegen, Nijmegen, The Netherlands, because it will be responsible for tracking any changes made manually to raw data. Any other data will be uploaded (after site-specific quality control) by the research team to the central database using encrypted mass storage devices. All information collected in the study will be coded with a unique pseudocode identifier (PSC) in such a way that participants cannot be identified from the corresponding data (directive 95/46/EC) and ensuring that the information collected for the study remains strictly confidential. Likewise, collected samples of biomaterial (i.e., blood and microbiota) will be labeled with the corresponding PSC. All patient-related data will be stored at the clinical sites in a database with password protection. A separate single database on the central server of VHIR will link the PSC to the participant’s personal data (including name, address, telephone details, and date of birth). Databases will be accessible only to the immediate research team at each site.

### Study monitoring

Proper conduct of data collection in the trial will be monitored via on-site visits of a monitoring staff member throughout the study; quality of data collected will further be monitored regularly by a statistical supervision team. After the first five participants per site are included, the quality of data (on item/trial level) per participant will be checked, aiming at detecting any error that may occur in the beginning of the project and to prevent these errors from recurring. After including participant numbers 5 to 10, the completeness and accuracy of the data on summary/scale level will be checked for all participants. Thereafter, data on a summary/scale level will be checked randomly for 1 in 5 participants. Outcomes of this data check will be written in an overview and reported to all research personnel. If adjustments to the database or procedures need to be made, these will be made as soon as possible. Moreover, the investigators will permit quality data checks, audits, and inspections by providing the sponsor direct access to source data and other documents (e.g., medical files) by request.

In each participating center, the PI will be responsible for the local ethics application, on-site training of clinicians, identification and recruitment of participants including randomization, data collection according to the study protocol, completion of the case report forms including answering queries, maintaining and updating the investigator site file, participation in monitoring, and reporting SAEs to the study PI in Barcelona. Each site will have regular meetings with their clinical team in order to ensure progress of the trial. As the leading coordinator center and with the aim of monitoring the study progress, the VHIR team will arrange telephone meetings with the local sites throughout the project on a regular basis.

Due to the need for research groups to share their data to maximize the value of each research contribution, to pool them to address research questions that require larger numbers, or to carry out meta-analyses, participants and, if applicable, their legal guardians will be asked whether they consent that their data (or portions of their data) will be shared in anonymized fashion with other research projects.

### Statistical analysis

Primary outcome measures will be analyzed by applying logistic regression analyses with type of treatment, diagnostic group (e.g., ADHD, BPD, or both), gender, and center as independent variables. A sample size of 180 participants will allow for detection of an odds ratio of 2.55 with 90% power, assuming a response rate of 20% for the CG at a significance level of 0.05. For simplification purposes, calculations will assume no effects of the covariates on the response rate. Secondary analyses will be performed on the secondary outcome measures assessed at the different time points. General linear models will be applied to examine effects of treatment group on continuous outcome variables while controlling for baseline assessment, gender, diagnostic group, and center. Categorical outcome measures will be investigated using generalized linear models. Treatment effects and their 95% confidence intervals will be reported. To investigate potential long-term effects of probiotics, data of the EG for 10 weeks will be analyzed. General linear models for repeated measurements will be applied to study changes from baseline to follow-up visit for this group, and missing data will be handled by the last observation carried forward method. Statistical significance will be defined at the 0.05 level.

Once the primary and secondary outcome measures results are obtained, all responsible investigators at all study sites will get access to the data to be able to reanalyze the data regarding specific additional research questions.

In case of an external request for replication, the respective statistical analysis will be provided by the trial statistician.

### Ethics and dissemination

Before the first subject is enrolled in the trial, all ethical and legal requirements will be met. The study protocol, participant information, and the respective consent form will be approved according to the respective local and national regulations at each of the participating centers before the start of the trial, and participants will be made aware of the investigational nature and the core aspects of the study as well. This study will be conducted in accordance with the principles of the Declaration of Helsinki (2008) and the Medical Research Involving Human Subjects Act (WMO). It will follow the principles of the Guideline for Good Clinical Practice (ICH GCP guideline E6), EU Directive 2001/20/EC, and applicable regulatory requirements and guidelines in the participating countries/regions. The handling of personal data will be done in accordance with the new General Data Protection Regulation that started in May 2018 in the European Union.

Trial findings will be reported to public and private healthcare providers, specific stakeholders such as policy makers, the medical community, or academic and commercial parties interested in therapy development, as well as the general public via publications, conferences, press releases, public talks, and the internet- (e.g., YouTube, Facebook, Twitter). All trial results will be reported in accordance with the Consolidated Standards of Reporting Trials (CONSORT) guidelines (www.consort-statement.org).

## Discussion

We have presented a design and protocol for an RCT of a nonpharmacological intervention—synbiotic treatment—for the reduction of impulsive and compulsive symptomatology in a sample of adult patients with ADHD and/or BPD. The PROBIA study is the first multinational RCT evaluating the effects of probiotics on cognitive function, impulsivity, and compulsivity in a large ADHD and/or BPD sample. Existing studies primarily addressing the effects of supplementation with probiotics in psychiatric disorders mostly come from animal studies or have been done in smaller samples. Moreover, to date, no RCT has evaluated the composition of gut microbiota in ADHD and/or BPD populations with a large sample size. Thus, whether the microbial community in these individuals is different from that in mentally healthy humans remains unknown.

Our study will also allow us to identify links between the microbiome and hallmark characteristics of ADHD and/or BPD patients (e.g., impulsivity, compulsivity, or aggressive behavior) and also to investigate whether dietary patterns and probiotics can induce alterations in the gut microbiota, which may mediate/moderate effects on these behaviors. Linking these data with blood biomarkers as well as genetic and epigenetic parameters will provide integrated mechanistic knowledge on diet/lifestyle–gut–brain–behavior relationships relevant to impulsivity and compulsivity. In order to create a study sample population that is highly representative of impulsivity, broad inclusion criteria will be applied, with most psychiatric comorbidities being accepted, allowing us to provide a better illustration of possible effects that could be expected if the intervention were introduced in a clinical setting. Understanding the microbiota would be important both for better comprehension of the biological bases that underlie the studied disorders and for the future development of new evidenced-based medications for these conditions.

## Trial status

This trial is registered with ClinicalTrials.gov (NCT03495375) and was first posted on 12 April 2018. The first participant gave consent on 22 February 2019 and was randomized on 27 February 2019. Recruitment is expected to be completed 1 April 2021. The most recent version of the protocol (V2.0) was approved by the Ethical Committee of the University Hospital Vall d’Hebron, Barcelona, Spain, on 12 April 2019 [PR (AG)311-2018].

## Supplementary information

**Additional file 1.** Standard Protocol Items: Recommendations for Interventional Trials (SPIRIT) 2013 checklist: recommended items to address in a clinical trial protocol and related documents.

## Data Availability

Not applicable.

## Abbreviations

24HDR: 24-h dietary recall
ADHD: Attention-deficit/hyperactivity disorder
ADHD-RS: Attention-Deficit/Hyperactivity Disorder Rating Scale
AE: Adverse event
ARI-S: Affective Reactivity Index–Self-Report
ASD: Autism spectrum disorder
BPD: Borderline personality disorder
CGI-I: Clinical Global Impression–Improvement Scale
CGI-S: Clinical Global Impression–Severity Scale,
CPT-II: Continuous Performance Test
DERS-16: Difficulties in Emotion Regulation Scale
DIVA 2.0: Diagnostic Interview for Adult ADHD, Second Edition
EG: Experimental group (probiotic)
GCP: Good clinical practice
IL: Interleukin
IQ: Intelligence quotient
LAB: Lactic acid bacteria
M.I.N.I.-Plus: Mini-International Neuropsychiatric Interview
PI: Principal investigator
ProMAS: Probabilistic Medication Adherence Scale
PSC: Pseudocode identifier
PUFA: Polyunsaturated fatty acid
RCT: Randomized controlled trial
SAE: Serious adverse event
SCID-II: Structured Clinical Interview for DSM-IV Axis II Disorders
SDQ: Strengths and Difficulties Questionnaire
SES: Socioeconomic status
SF: Synbiotic 2000 Forte
SUSAR: Suspected unexpected serious adverse reaction
UPPS-P: Urgency, Premeditation (lack of), Perseverance (lack of), Sensation Seeking, Positive Urgency, Impulsive Behavior Scale
VHIR: Vall d’Hebron Research Institute
WAIS: Wechsler Adult Intelligence Scale
Y-BOCS: Yale-Brown Obsessive Compulsive Scale

## References

[1] Fineberg NA, Chamberlain SR, Goudriaan AE, Stein DJ, Vandershuren LJ, Gillan CM (2014). New developments in human neurocognition: clinical, genetic, and brain imaging correlates of impulsivity and compulsivity. CNS Spectr.

[2] Fineberg NA, Potenza MN, Chamberlain SR, Berlin HA, Menzies L, Bechara A (2010). Probing compulsive and impulsive behaviors, from animal models to endophenotypes: a narrative review. Neuropsychopharmacology.

[3] Potenza MN (2007). To do or not to do? The complexities of addiction, motivation, self-control, and impulsivity. Am J Psychiatry.

[4] Chamberlain SR, Sahakian BJ (2007). The neuropsychiatry of impulsivity. Curr Opin Psychiatry.

[5] Chamberlain SR, Fineberg NA, Blackwell AD, Robbins TW, Sahakian BJ (2006). Motor inhibition and cognitive flexibility in obsessive-compulsive disorder and trichotillomania. Am J Psychiatry.

[6] Wykes T, Haro JM, Belli SR, Obradors-Tarragó C, Arango C, Ayuso-Mateos JL (2015). Mental health research priorities for Europe. Lancet Psychiatry.

[7] Polanczyk G, de Lima MS, Horta BL, Biederman J, Rohde LA (2007). The worldwide prevalence of ADHD: a systematic review and metaregression analysis. Am J Psychiatry.

[8] Dalsgaard S, Leckman JF, Mortensen PB, Nielsen HS, Simonsen M (2015). Effect of drugs on the risk of injuries in children with attention deficit hyperactivity disorder: a prospective cohort study. Lancet Psychiatry.

[9] Sarris J, Logan AC, Akbaraly TN, Amminger GP, Balanzá-Martínez V, Freeman MP (2015). Nutritional medicine as mainstream in psychiatry. Lancet Psychiatry.

[10] Willatts P (2018). Effects of nutrition on the development of higher-order cognition. Nestle Nutr Inst Workshop Ser.

[11] Mohajeri MH, Wittwer J, Vargas K, Hogan E, Holmes A, Rogers PJ (2015). Chronic treatment with a tryptophan-rich protein hydrolysate improves emotional processing, mental energy levels and reaction time in middle-aged women. Br J Nutr.

[12] Mohajeri MH, Troesch B, Weber P (2015). Inadequate supply of vitamins and DHA in the elderly: implications for brain aging and Alzheimer-type dementia. Nutrition.

[13] Malinin NL, West XZ, Byzova TV (2011). Oxidation as “the stress of life”. Aging (Albany NY).

[14] Hibbeln JR (2001). Seafood consumption and homicide mortality: a cross-national ecological analysis. World Rev Nutr Diet.

[15] Janssen CI, Jansen D, Mutsaers MP, Dederen PJ, Geenen B, Mulder MT (2016). The effect of a high-fat diet on brain plasticity, inflammation and cognition in female ApoE4-knockin and ApoE-knockout mice. PLoS One.

[16] Stevenson RJ, Prescott J (2014). Human diet and cognition. Wiley Inerdiscip Rev Cogn Sci.

[17] Rijlaardam J, Cecil CA, Walton E, Mesirow MS, Relton CL, Gaunt TR (2017). Prenatal unhealthy diet, insulin-like growth factor 2 gene (IGF2) methylation, and attention deficit hyperactivity disorder symptoms in youth with early-onset conduct problems. J Child Psychol Psychiatry.

[18] Wald DS, Kasturiratne A, Simmonds M (2010). Effect of folic acid, with or without other B vitamins, on cognitive decline: meta-analysis of randomized trials. Am J Med.

[19] Dangour AD, Andreeva VA, Sydenham E, Uauy R (2012). Omega 3 fatty acids and cognitive health in older people. Br J Nutr.

[20] Mazereeuw G, Lanctot KL, Chau SA, Swardfager W, Herrmann N (2012). Effects of ω-3 fatty acids on cognitive performance: a meta-analysis. Neurobiol Aging.

[21] Clarke R, Bennet D, Parish S, Lewington S, Skeaff M, Eussen SJ (2014). Effects of homocysteine lowering with B vitamins on cognitive aging: meta-analysis of 11 trials with cognitive data on 22,000 individuals. Am J Clin Nutr.

[22] Massee LA, Ried K, Pase M, Travica N, Yoganatahn J, Scholey A (2015). The acute and sub-chronic effects of cocoa flavanols on mood, cognitive and cardiovascular health in young healthy adults: a randomized, controlled trial. Front Pharmacol.

[23] Ursell LK, Metcalf JL, Wegener-Partrey L, Knight R (2012). Defining the human microbiome. Nutr Rev.

[24] Rogers GB, Keating DJ, Young RL, Wong ML, Licinio J, Wesselingh S (2016). From gut dysbiosis to altered brain function and mental illness: mechanisms and pathways. Mol Psychiatry.

[25] Parashar A, Udayabanu M (2017). Gut microbiota: implications in Parkinson’s disease. Parkinsonism Relat Disord.

[26] Borgo F, Riva A, Benetti A, Casiraghi MC, Bertelli S, Garbossa S (2017). Microbiota in anorexia nervosa: the triangle between bacterial species, metabolites and psychological tests. PLoS One.

[27] Felice VD, O’Mahony SM (2017). The microbiome and disorders of the central nervous system. Pharmacol Biochem Behav.

[28] O’Mahony SM, Clarke G, Dinan TG, Cryan JF (2017). Early-life adversity and brain development: is the microbiome a missing piece of the puzzle?. Neuroscience.

[29] Strati F, Cavalieri D, Albanese D, De Felice C, Donati C, Hayek J (2016). Altered gut microbiota in Rett syndrome. Microbiome.

[30] Desbonnet L, Clarke G, Shanahan F, Dinan TG, Cryan JF (2014). Microbiota is essential for social development in the mouse. Mol Pyschiatry.

[31] Tomova A, Husarova V, Lakatosova S, Bakos J, Vikova B, Babinska K (2015). Gastrointestinal microbiota in children with autism in Slovakia. Physiol Behav.

[32] Wang L, Conlon MA, Christophersen CT, Sorich MJ, Angley MT (2014). Gastrointestinal microbiota and metabolite biomarkers in children with autism spectrum disorders. Biomark Med.

[33] Finegold SM, Downes J, Summanen PH (2012). Microbiology of regressive autism. Anaerobe.

[34] Finegold SM, Dowd SE, Gontchahrova V, Liu C, Henley KE, Wolcott RD (2010). Pyrosequencing study of fecal microflora of autistic and control children. Anaerobe.

[35] Parracho HM (2005). Differences between the gut microflora of children with autistic spectrum disorders and that of healthy children. J Med Microbiol.

[36] Aarts E, Ederveen THA, Naaijen J, Zwiers MP, Boekhorst J, Timmerman HM (2017). Gut microbiome in ADHD and its relation to neural reward anticipation. PLoS One.

[37] Petra AI, Panagiotidou S, Hatziagelaki E, Stewart JM, Conti P, Theoharides TC (2015). Gut–microbiota–brain axis and its effect on neuropsychiatric disorders with suspected immune dysregulation. Clin Ther.

[38] Galley JD, Bailey MT (2014). Impact of stressor exposure on the interplay between commensal microbiota and host inflammation. Gut Microbes.

[39] Cryan JF, Dinan TG (2012). Mind-altering microorganisms: the impact of the gut microbiota on brain and behavior. Nat Rev Neurosci.

[40] Ming X, Chen N, Ray C, Brewer G, Kornitzer J, Steer RA (2018). A gut feeling: a hypothesis of the role of the microbiome in attention-deficit/hyperactivity disorders. Child Neurol Open.

[41] Mowry EM, Glenn JD (2018). The dynamics of the gut microbiome in multiple sclerosis in relation to disease. Neurol Clin.

[42] Strati F, Cavalieri D, Albanese D, De Felice C, Donati C, Hayek J (2017). New evidences on the altered gut microbiota in autism spectrum disorders. Microbiome.

[43] Stirpe P, Hoffman M, Badiali D, Colosimo C (2016). Constipation: an emerging risk factor for Parkinson’s disease?. Eur J Neurol.

[44] Wang YP, Chen YT, Tsai CF, Li SY, Luo SY, Wang SJ (2014). Short-term use of serotonin reuptake inhibitors and risk of upper gastrointestinal bleeding. Am J Psychiatry.

[45] Kang V, Wagner GC, Ming X (2014). Gastrointestinal dysfunction in children with autism spectrum disorders. Autism Res.

[46] McKeown C, Hisle-Gorman E, Eide M, Gorman GH, Nylund CM (2013). Association of constipation and fecal incontinence with attention-deficit/hyperactivity disorder. Pediatrics.

[47] Motil KJ, Caeg E, Barrish JO, Geerts S, Lane JB, Percy AK (2012). Gastrointestinal and nutritional problems occur frequently throughout life in girls and women with Rett syndrome. J Pediatr Gastroenterol Nutr.

[48] Knowles SR, Nelson EA, Palombo EA (2008). Investigating the role of perceived stress on bacterial flora activity and salivary cortisol secretion: a possible mechanism underlying susceptibility to illness. Biol Psychol.

[49] Mayer EA (2011). Gut feelings: the emerging biology of gut-brain communication. Nat Rev Neurosci.

[50] Dinan TG, Cryan JF (2017). Gut instincts: microbiota as a key regulator of brain development, ageing and neurodegeneration. J Physiol.

[51] Diaz Heijtz R, Wang S, Anuar F, Qian Y, Björkholm B, Samuelsson A (2011). Normal gut microbiota modulates brain development and behavior. Proc Natl Acad Sci U S A.

[52] Felix KM, Tahsin S, Wu HJ (2018). Host-microbiota interplay in mediating immune disorders. Ann N Y Acad Sci.

[53] Yadav SK, Boppana S, Ito N, Mindur JE, Mathay MT, Patel A (2017). Gut dysbiosis breaks immunological tolerance toward the central nervous system during young adulthood. Proc Natl Acad Sci U S A.

[54] Mandl T, Marsal J, Olsson P, Ohlsson B, Andreasson K (2017). Severe intestinal dysbiosis is prevalent in primary Sjögren’s syndrome and is associated with systemic disease activity. Arthritis Res Ther.

[55] Poletti S, de Wit H, Mazza E, Wijkhuijs AJM, Locatelli C, Aggio V (2017). Th17 cells correlate positively to the structural and functional integrity of the brain in bipolar depression and healthy controls. Brain Behav Immun.

[56] Cenit MC, Nuevo IC, Codoñer-Franch P, Dinan TG, Sanz Y (2017). Gut microbiota and attention deficit hyperactivity disorder: new perspectives for a challenging condition. Eur Child Adolesc Psychiatry.

[57] Anand D, Colpo GD, Zeni G, Zeni CP, Teixeira AL (2017). Attention-deficit/hyperactivity disorder and inflammation: what does current knowledge tell us? A systematic review. Front Psychiatry.

[58] Miyazaki C, Koyama M, Ota E, Swa T, Mlunde LB, Amiya RM (2017). Allergic diseases in children with attention deficit hyperactivity disorder: a systematic review and meta-analysis. BMC Psychiatry.

[59] Shans JV, Cicek R, de Vries TW, Hak E, Hoekstra PJ (2017). Association of atopic diseases and attention-deficit/hyperactivity disorder: a systematic review and meta-analysis. Neurosci Biobehav Rev.

[60] Nielsen PR, Benros ME, Dalsgaard S (2017). Associations between autoimmune diseases and attention-deficit/hyperactivity disorder: a nationwide study. J Am Acad Child Adolesc Psychiatry.

[61] Hegvik TA, Instanes JT, Haavik J, Klungsoyr K, Engeland A (2018). Associations between attention-deficit/hyperactivity disorder and autoimmune diseases are modified by sex: a population-based cross-sectional study. Eur Child Adolesc Psychiatry.

[62] Donfrancesco R, Nativio P, Di Benedetto A, Villa MP, Andriola E, Melegari MG, et al. Anti-Yo antibodies in children with ADHD: first results about serum cytokines. J Atten Disord. 10.1177/1087054716643387. 10.1177/1087054716643387 27095560

[63] Allred EN, Dammann O, Fichorova RN, Hooper SR, Hunter SJ, Joseph RM (2017). Systemic inflammation during the first postnatal month and the risk of attention deficit hyperactivity disorder characteristics among 10-year-old children born extremely preterm. J NeuroImmune Pharmacol.

[64] Rand KM, Austin NC, Inder TE, Bora S, Woodward LJ (2016). Neonatal infection and later neurodevelopmental risk in the very preterm infant. J Pediatr.

[65] Toto M, Margari F, Simone M, Craig F, Petruzzelli MG, Tafuri S (2015). Antibasal ganglia antibodies and antistreptolysin O in noncomorbid ADHD. J Atten Disord.

[66] Giana G, Romano E, Porfirio MC, D’Ambrosio R, Giovinazzo S, Troianiello M (2015). Detection of auto-antibodies to DAT in the serum: interactions with DAT genotype and psychostimulant therapy for ADHD. J Neuroimmunol.

[67] Mitchell RH, Goldstein BI (2014). Inflammation in children and adolescents with neuropsychiatric disorders: a systematic review. J Am Acad Child Adolesc Psychiatry.

[68] Wei H, Alberts I, Li X (2013). Brain IL-6 and autism. Neuroscience.

[69] Williams NT (2010). Probiotics. Am J Health Syst Pharm.

[70] Franco-Robles E, López MG (2015). Implications of fructans in health: immunomodulatory and antioxidant mechanisms. ScientificWorldJournal.

[71] Slykerman RF, Kang J, Van Zyl N, Barthow C, Wickens K, Stanley T (2018). Effect of early probiotic supplementation on childhood cognition, behaviour and mood: a randomized, placebo-controlled trial. Acta Paediatr.

[72] Kane L, Kinzel J (2018). The effects of probiotics on mood and emotion. JAAPA.

[73] Reber SO, Siebler PH, Donner NC, Morton JT, Smith DG, Kopelman JM (2016). Immunization with a heat-killed preparation of the environmental bacterium *Mycobacterium vaccae* promotes stress resilience in mice. Proc Natl Acad Sci U S A.

[74] Steenbergen L, Sellaro R, van Hemert S, Bosch JA, Colzato LS (2015). A randomized controlled trial to test the effect of multispecies probiotics on cognitive reactivity to sad mood. Brain Behav Immun.

[75] Tillisch K, Labus J, Kilpatrick L, Jiang Z, Stains J, Ebrat B (2013). Consumption of fermented milk product with probiotic modulates brain activity. Gastroenterology.

[76] Messaoudi M, Lalonde R, Violle N, Javelot H, Desor D, Nejdi A (2011). Assessment of psychotropic-like properties of a probiotic formulation (*Lactobacillus helveticus* R0052 and *Bifidobacterium longum* R0175) in rats and human subjects. Br J Nutr.

[77] Gareau MG, Wine E, Rodrigues DM (2011). Bacterial infection causes stress-induced memory dysfunction in mice. Gut.

[78] Pärtty A, Kalliomäki M, Wacklin P, Salminen S, Isolauri E (2015). A possible link between early probiotic intervention and the risk of neuropsychiatric disorders later in childhood: a randomized trial. Pediatr Res.

[79] Yousefi B, Eslami M, Ghasemian A, Kokhaei P, Salek Farrokhi A, Drabi N (2018). Probiotics importance and their immunomodulatory properties. J Cell Physiol.

[80] Mardani F, Mahmoudi M, Esmaeli SA, Khorasani S, Tabasi N, Rastin M (2018). In vivo study: Th1-Th17 reduction in pristane-induced systemic lupus erythematosus mice after treatment with tolerogenic *Lactobacillus* probiotics. J Cell Physiol.

[81] Tan M, Zhu JC, Du J, Zhang LM, Yin HH. Effects of probiotics on serum levels of Th1/Th2 cytokine and clinical outcomes in severe traumatic braininjured patients: a prospective randomized pilot study. Crit Care.2011;15(6):R290. 10.1186/cc10579 PMC3388628 22136422

[82] Tanabe S. The effect of probiotics and gut microbiota on Th17 cells. Int Rev Immunol.2013;32(5-6):511-25.. 10.3109/08830185.2013.839665 24094077

[83] Chan AW, Tetzlaff JM, Gotzsche PC, Altman DG, Mann H, Berlin JA, et al. SPIRIT 2013 explanation and elaboration : guidance for protocols of clinical trials. BMJ. 2013; 346:e7586. 10.1136/bmj.e7586 PMC3541470 23303884

